# The Diagnostic Accuracy of First-Year Ophthalmology Residents on Call: Considerations for Postgraduate Year 2 (PGY-2) Standardizations of Call Structure

**DOI:** 10.7759/cureus.59206

**Published:** 2024-04-28

**Authors:** Marium Hashemi, Shawyon C Rohani, Fabliha A Mukit, Shane Marsili, Emmanuel Sarmiento, Eric J Zhang, Stephen C Dryden, Brian M Jerkins, Brian T Fowler

**Affiliations:** 1 Ophthalmology, Hamilton Eye Institute, Memphis, USA; 2 Ophthalmology, University of Kentucky, Lexington, USA; 3 Ophthalmology, Cook County Health, Chicago, USA; 4 Ophthalmology, University of Missouri, Columbia, USA; 5 Ophthalmology, Tennessee Oculoplastics, Nashville, USA

**Keywords:** pgy-2, on call, primary call, diagnostic accuracy, standardization, resident, supervision, curriculum

## Abstract

Background

Ophthalmology is a unique specialty with limited exposure during medical school. To improve the transition to ophthalmology residency, the Accreditation Council for Graduate Medical Education (ACGME) announced in 2017 that all ophthalmology residency programs would move to a combined post-graduate year (PGY) 1 year with mandatory integration by 2023. Currently, there are no standardized guidelines from the American Board of Ophthalmology (ABO) or the Accreditation Council for Graduate Medical Education (ACGME) to address ophthalmology resident competence prior to becoming the primary contact for inpatient and emergency room (ER) consultations as a PGY-2. Novice residents may not be equipped to accurately diagnose vision or life-threatening ocular conditions. A balance between resident autonomy and supervision is required for proper training without increasing patient morbidity and mortality.

Objective

This study’s objective is to examine the diagnostic accuracy of PGY-2 ophthalmology non-integrated residents on call to standardize supervision requirements (through buddy-call) prior to initiating indirectly supervised calls.

Methods

All inpatient and ER ophthalmology consults for the first seven weeks of the year evaluated by PGY-2 (junior) residents were supervised and graded as “correct” or “incorrect” by PGY-4 (senior) residents.

Results

One hundred forty-eight consults were seen over 30 call days over a period of seven weeks (4.93 consults per call). The percentage of correct diagnoses increased with each successive week (R^2^ = 0.9581; correlation = 0.979). The greatest percent increase of correctly diagnosed encounters was between weeks 2 and 3 (19.14%) correlating to call numbers 10-16 and 45-68 patient encounters. The mean percent accuracy surpassed 70% during weeks 3-4, and improvement continued to week 7. High-acuity diagnoses were identified consistently 100% of the time from week 5 onward.

Conclusion

Our analysis indicated that diagnostic accuracy was greater than 70% between weeks 3 and 4 with high-acuity diagnostic accuracy reaching 100% at week 5. It can be postulated that optimal direct senior resident supervision is needed for at least 3-5 weeks before transitioning to indirectly supervised calls by PGY-2 residents. This standardization would allow junior residents to acquire sufficient clinical experience to accurately make a diagnosis and prevent patient morbidity. Further research nationally is necessary prior to creating a standardized call structure for PGY-2 residents especially with the newly mandatory integrated ophthalmology residency programs.

## Introduction

Exposure to ophthalmology is limited prior to starting formal residency training resulting in a steep learning curve during the transition period. Although an integrated transition year has become the new standard for ophthalmology residency [1,2], there is still significant variability in internship requirements, which affects the baseline knowledge of post-graduate year (PGY) 2 residents nationwide [3]. Currently, there is no standardized national surgical curriculum for ophthalmology [4]. While several studies support the idea of standardizing the approach to ophthalmology training [5-7], the American Academy of Ophthalmology’s (AAO) Basic Clinical and Science Course is the only consistent national curriculum for ophthalmology in the United States [8]. Novice residents may lack sufficient experience in utilizing appropriate equipment and techniques that are necessary for the diagnostic accuracy of ocular conditions. Certain diagnoses such as assessing for corneal abrasions have a more straightforward management algorithm to learn; however, the evaluation for diagnosing high-acuity cases such as orbital fracture with entrapped extraocular muscle(s), macula-on retinal detachment, endophthalmitis, orbital cellulitis, or a ruptured globe requires a more advanced knowledge base and examination skillset.

Of the many difficult aspects of beginning ophthalmology residency training, a primary call may be considered one of the most strenuous experiences with a steep learning curve in a high-pressure environment. With limited knowledge and diagnostic abilities consistent with their level of training, PGY-2 residents are expected to identify ocular pathology, triage patients, and develop a treatment plan with modest supervision. Throughout the nation, residency programs use various approaches to prepare new ophthalmology residents for taking primary calls. A cross-sectional survey given to program directors and first-year residents in 2016 and 2017 evaluated first-year call structure and preparation. Of those who responded, 97.4% of the residents reported a buddy-call system, and 60.8% reported utilizing a buddy-call system between three and eight weeks. Most residents reported taking independent primary calls between four and eight weeks (71.9%) [9]. Within radiology literature, it was found that the buddy-call system and tiered call structure proved helpful for junior residents transitioning to independent calls [10]. In our discussion with multiple chief residents at various programs across the country, the call structure for junior residents included buddy-call (a senior resident must see every patient with the junior), must-call (where every case must be discussed with a senior resident), backup call (senior only discusses or comes in for select cases), and independent call.

Call is an essential aspect of residency training that serves to prepare physicians for autonomous decision-making and independent practice. The supervision of junior residents can be a strong educational experience but, with prolonged supervision, can cause a decrease in confidence and poor independent decision-making skills. Conversely, the lack of oversight of inexperienced PGY-2 residents who serve as the primary contact for inpatient and emergency room (ER) consultations may result in increased patient morbidity. While consultations may be as benign as refractive error, requests to evaluate patients from inpatient and emergency providers can range from vision-threatening to life-threatening conditions that novice residents may not be equipped to diagnose [11,12]. In a 2008 study by Statham et al., it was found that general practitioners, emergency room physicians, and optometrists made accurate ocular diagnoses 25.9%, 4.9%, and 48.2% of the time, respectively. Patients had adverse outcomes from misdiagnosis and mismanagement 11.6% of the time [13]. Most primary care providers do not receive adequate training to assess ocular diseases, and medical school education in ophthalmic disease is sparse; therefore, PGY-2 residents serve as the first-line physicians for ocular consultations from July when all specialties transition to a new year with new residents. A retrospective study in 2010 revealed fatal medical errors increased by 10% during the month of July [14]. Additionally, a study in 2017 reported double the medical errors for pediatric residents in July compared to the months of May, June, and August [15]. These studies highlight the risk of medical errors in specialties that medical students have exposure to compared to a field such as ophthalmology. Though there are currently no medical error-related studies for ophthalmology residents in the literature, there is concern that the medical error rate may be higher in ophthalmology candidates due to limited exposure in medical school.

In this study, we compared the diagnostic accuracy of non-integrated PGY-2 residents’ inpatient and ER consultations to the PGY-4 residents during the transition period from internship to categorical ophthalmology residency. We evaluated the length of time required to reach a 70% diagnostic accuracy, the impact of the primary clinical rotation on reaching the diagnostic threshold, and the time required to correctly diagnose high-acuity cases consistently. Our goal was to develop a standardized recommendation that would adequately prepare PGY-2 residents for indirectly supervised calls while providing appropriate care to patients.

## Materials and methods

Study setting and participant selection

This was a prospective cohort study conducted at the Hamilton Eye Institute in Memphis, Tennessee, from July 2019 to August 2021 using stratified sampling (all PGY-2 residents and all PGY-4 residents). All inpatient and emergency room consults that were performed by PGY-2 (junior) residents during the initial seven weeks of the academic year were directly supervised by PGY-4 (senior) residents.

Assessment protocol

After examining a patient, junior residents were required to present the information they gathered from their encounter, including the patient’s history, their physical examination findings, the resident’s differential diagnosis, their assessment, and their plan for managing the patient to their senior counterparts. The senior resident then evaluated the encounter and, based on the junior resident’s final diagnosis and assessment, graded the encounter as either “correct” or “incorrect.” Diagnoses that were partially incorrect or incomplete were graded as incorrect.

Statistical analysis

The statistical analysis of the grades for the encounters was performed using Microsoft Excel (Microsoft Corp., Redmond, WA). A linear regression analysis was performed with respect to correct diagnoses and the week of the academic year in order to explore the potential relationship between diagnostic accuracy and the progression of clinical proficiency over time as PGY-2 year progressed.

To more thoroughly observe the factors affecting diagnostic accuracy, further stratification of the data was performed based on the week of training each resident was in, the type of rotation the resident was completing at the time, and whether the diagnosis was considered high acuity. The first-year resident rotations, which each had distinct responsibilities and patient populations, were as follows: residents who worked in the resident clinic (two residents per rotation) and were responsible for daytime consults at a regional level 1 trauma hospital and level 2 university hospital, retina service residents who were responsible for daytime consults at a community level 2 hospital, Veterans Administration (VA) service residents who were responsible for daytime consults at a VA hospital, and glaucoma residents who had no daytime consult responsibility. High-acuity diagnoses were determined by the program director and included those conditions that could be potentially life- or vision-threatening. Overall, there were 14 high-acuity pathologies including retinal detachment, acute angle-closure glaucoma, and infectious keratitis. In total, our project analyzed the data from 223 total consults seen by five junior residents over the course of seven weeks. The statistical significance of this study was set to p < 0.05.

## Results

Diagnostic accuracy over time

**Figure 1:**
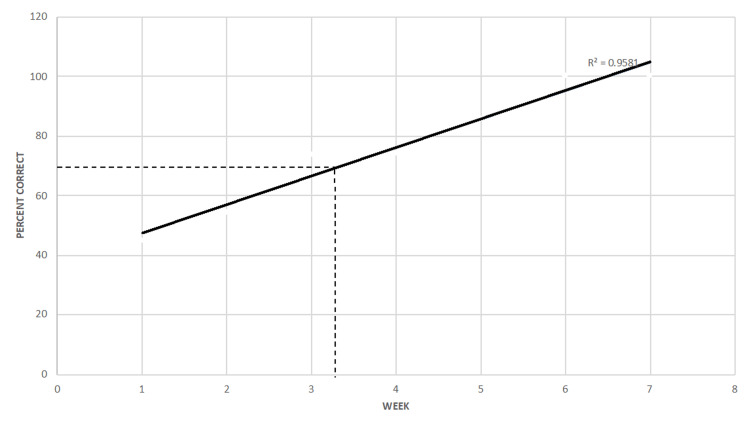
First-Year Resident Percentage of Correct Diagnosis During Buddy-Call

Of the 223 total consults seen over 47 call days since the beginning of the academic year, 148 consults were seen by PGY-2 residents on 30 separate call days (4.93 consults per call). From week 1 to week 7, the percentage of correct diagnoses increased (R^2^ = 0.96; correlation = 0.98; p = 0.0007). The largest increase in the percentage of correct diagnoses was from weeks 2 to 3 (19.14%), which corresponds to approximately 10-16 call days and 45-68 patient encounters. All PGY-2 residents crossed the threshold of 70% correct diagnoses by week 3 and continued to improve their diagnostic accuracy through week 7. Residents achieved 70% diagnostic accuracy between week 3 and week 4 (Table 1 and Figure 1).

**Table 1:** Diagnostic Accuracy Over Time

Week	Correct	Incorrect	Percentage Correct (%)	Percentage Change (%)
1	9	11	45.00	Not Applicable
2	12	10	54.55	9.55
3	14	5	73.68	19.14
4	23	8	74.19	0.51
5	18	3	85.71	11.52
6	23	0	100.00	14.29
7	14	0	100.00	0.00

Diagnostic accuracy based on primary rotation

Resident diagnostic accuracy was also evaluated based on their primary rotation during their PGY-2 year. Resident clinic residents surpassed the 70% diagnostic accuracy threshold in weeks 2 and 3. The VA, glaucoma/pediatrics, and retina service residents each surpassed the 70% diagnostic accuracy threshold in week 2, week 4, and week 5, respectively (Table 2).

**Table 2:** Diagnostic Accuracy Based on Primary Rotation VAMC: Veterans Administration Medical Center

Rotation	Percentage Accuracy Week 1 (%)	Percentage Accuracy Week 2 (%)	Percentage Accuracy Week 3 (%)	Percentage Accuracy Week 4 (%)	Percentage Accuracy Week 5 (%)	Percentage Accuracy Week 6 (%)	Percentage Accuracy Week 7 (%)
Resident Clinic	50.00	63.00	83.00	73.00	100.00	100.00	100.00
Resident Clinic	67.00	100.00	-	82.00	71.00	100.00	100.00
Glaucoma/Pediatrics	25.00	-	89.00	100.00	-	-	-
Retina	-	33.00	25.00	40.00	75.00	100.00	-
VAMC	44.00	100.00	-	-	100.00	100.00	-
Mean Percentage Accuracy	46.50	74.00	66.00	74.00	87.00	100.00	100.00

Diagnostic accuracy of high-acuity cases

**Figure 2:**
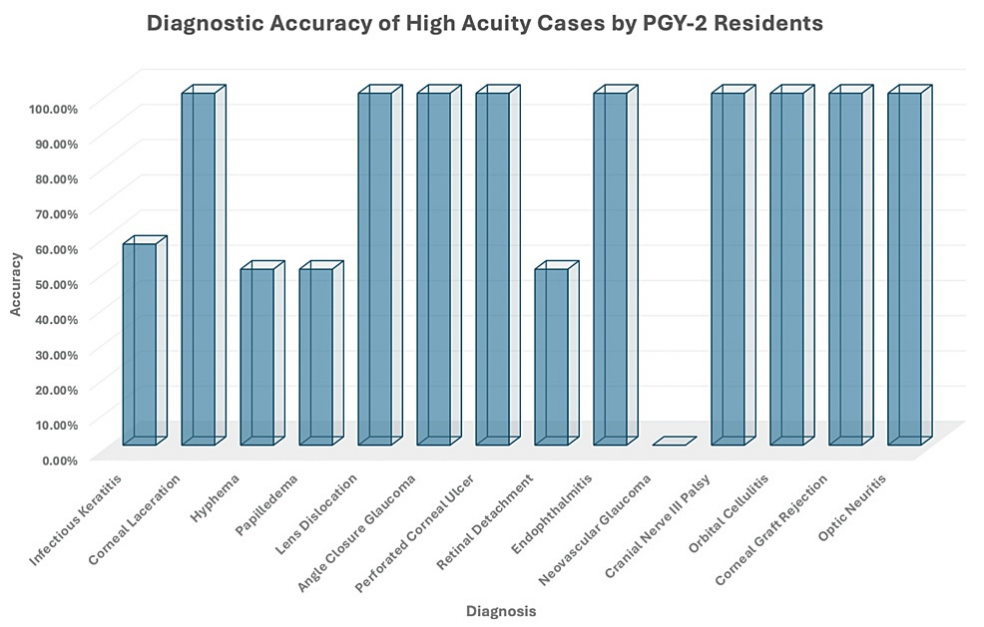
Diagnostic Accuracy of High-Acuity Cases by PGY-2 Residents PGY-2: postgraduate year 2

**Figure 3:**
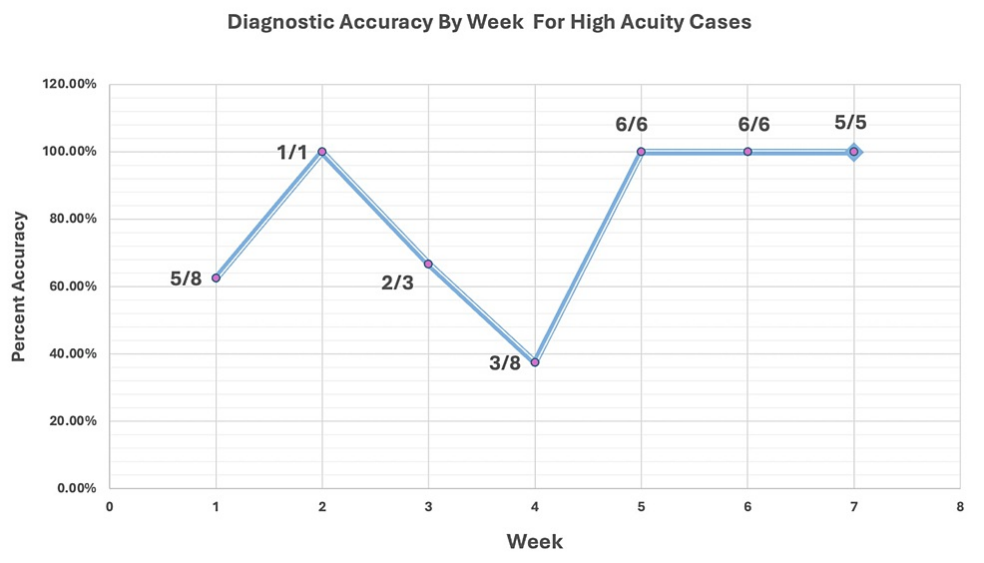
Diagnostic Accuracy by Week for High-Acuity Cases Each data point per week also includes a fraction to show the number of correct diagnoses made out of the total number of high-acuity cases that week. High-acuity diagnoses were determined by the program director and included conditions that could be potentially life- or vision-threatening with delayed evaluation and management. High-acuity diagnoses included infectious keratitis, corneal laceration, hyphema, papilledema, lens dislocation, angle-closure glaucoma, perforated corneal ulcer, retinal detachment, endophthalmitis, neovascular glaucoma, cranial nerve III palsy, orbital cellulitis, corneal graft rejection, and optic neuritis

Of the 148 consults received by the PGY-2 residents, there were 68 different diagnoses. Of them, 14 cases were flagged as high-risk vision loss diagnoses. These high-acuity diagnoses are important to recognize due to the potential vision- and life-threatening complications with delayed evaluation and management. Accurate diagnosis for each high-acuity case was as follows (the number of correct diagnoses/total number of diagnoses): infectious keratitis (4/7), corneal laceration (8/8), hyphema (3/6), papilledema (1/2), lens dislocation (2/2), angle-closure glaucoma (2/2), perforated corneal ulcer (2/2), retinal detachment (1/2), endophthalmitis (1/1), neovascular glaucoma (0/1), cranial nerve (CN) III palsy (1/1), orbital cellulitis (1/1), corneal graft rejection (1/1), and optic neuritis (1/1) (Figure 2). The analysis of the diagnostic accuracy of high-acuity cases per week reached 100% accuracy at week 2, with consistent 100% accuracy reached in week 5 onward. (Figure 3).

## Discussion

Each year, new ophthalmology residents face a difficult transition period in which they need to rapidly develop strong diagnostic abilities and quickly prepare for indirectly supervised calls. To provide efficient and accurate care to patients in need, PGY-2 residents have limited time to acquire the technical skillset and knowledge of ocular disease processes. The breadth of ophthalmic consultations PGY-2 residents are responsible for can range anywhere from as benign as a refractive error to as high stakes as CN III palsy with pupillary involvement with an imminent posterior communicating artery aneurysm that can result in death. Previously, a retrospective chart review evaluating ophthalmology resident on-call performance showed that 57.04% of consults were borderline to unsatisfactory in PGY-2 consultations compared to 35.71% of PGY-3 consults [16]. This provides insight into how diagnostic performance improves significantly with exposure and experience during ophthalmology residency.

In our study, diagnostic accuracy in PGY-2 residents improved considerably with increasing ophthalmic clinical experience and exposure. The ophthalmology residency site in this study begins PGY-2 year with a two-week preparatory course without any clinical or on-call duties. After this dedicated lecture and wet laboratory schedule, PGY-2 residents begin their clinical rotation and buddy-call schedule. Over a seven-week period, the junior residents were on call for 30 days and saw 148 consults directly with a senior resident. Residents were able to make the correct diagnosis 45% of the time during the first week with the highest improvement between week 2 and week 3 (19.14%). We determined that diagnostic accuracy of over 70% would be appropriate for a PGY-2 level, as senior residents and attending physicians are always available to discuss more difficult consultations. This threshold was surpassed during week 3 and continued to improve to week 6 when 100% diagnostic accuracy was achieved.

Additionally, we evaluated diagnostic accuracy based on each resident’s designated rotation when starting the PGY-2 year. In this residency program, two PGY-2 residents rotate through the resident clinic during a single 10-week block. They are responsible for daily outpatient clinics and all consultations from the university hospital and the level 1 trauma center. Ophthalmology exposure is the highest on this rotation as residents are required to independently work up all patients without any technician support. We postulate that this is the reason that the diagnostic accuracy of the residents on this rotation was the first to surpass the diagnostic accuracy of 70% threshold at weeks 2 and 3. At the VA, one PGY-2 resident rotates during each block. They experience the highest outpatient clinic volume of all rotations and are not required to have direct supervision when managing patients. However, there is full-time technical support for workup, and PGY-2 residents have minimal inpatient consultation burden. The VA resident achieved the 70% threshold at week 2. The glaucoma/pediatric rotation includes a more specific subset of patients without any daytime consultation responsibilities. The resident on this rotation surpassed the 70% threshold at week 3. The retina rotation has the least volume of patient encounters, predominantly limited to a single community hospital consultations, which is why we postulate that this resident achieved sufficient diagnostic accuracy last, at week 5.

In our study, we also evaluated the ability of residents to correctly diagnose high-acuity cases during their overnight call consultations. The high-acuity diagnosis was determined as cases that require 100% accuracy due to potentially blinding and life-threatening outcomes in the short term without appropriate evaluation and management. Of the 14 diagnoses selected by the program director as high acuity, nine were correctly identified 100% of the time (corneal laceration, lens dislocation, angle-closure glaucoma, perforated corneal ulcer, endophthalmitis, cranial nerve III palsy, orbital cellulitis, corneal graft rejection, and optic neuritis). Five high-acuity cases were diagnosed incorrectly partially or 100% of the time (neovascular glaucoma, infectious keratitis, hyphema, papilledema, and retinal detachment) (Figure 2). Diagnostic accuracy for high-acuity cases began at 62.5% in week 1 and reached 100% by week 5. In week 2, a 100% correct diagnosis was also reached; however, only one high-acuity case was seen during the entire seven-day period (infectious keratitis). Based on the overall diagnostic accuracy of infectious keratitis, this 100% accuracy in week 2 holds less significance as the diagnosis of infectious keratitis was identified correctly only four out of seven times in the seven-week period. Diagnostic accuracy was the lowest in week 4 during which only three high-acuity cases were correctly identified out of eight and included a missed diagnosis of infectious keratitis and a hyphema.

Based on our findings, a five-week buddy-call system would result in an optimal call structure for PGY-2 residents resulting in guarded dependence, decreasing senior call burden, and ensuring patient safety. In addition, while the two-week preparatory course may have offered the same baseline education to all new residents in training, there was a discrepancy in the time required for each resident to meet the 70% accuracy competency target. It is also important to note that the rotation a resident started in their PGY-2 year affected their performance on call significantly. This leads us to consider implementing clinical responsibilities as a preparatory measure prior to the start of the PGY-2 year.

In addition to standardizing call structure for new PGY-2 residents, the implementation of integrated ophthalmology residency programs throughout the United States will most likely improve PGY-2 residents’ capabilities and blunt the steep learning curve during the July transition period. Prior to the Accreditation Council for Graduate Medical Education’s (ACGME) requirement of an integrated ophthalmology residency program by 2023, a white paper in the American Academy of Ophthalmology (AAO) identified that the American Board of Ophthalmology (ABO) allowed internships in any field before beginning ophthalmology training. This resulted in highly variable intern year experiences among a single class of residents, as well as between programs. Integrated internships offer the opportunity to initiate ophthalmic training for up to six additional months, allowing a steady increase in knowledge and clinic acumen prior to the start of the PGY-2 year [2]. One recent study found that 61.5% of program directors indicated that PGY-1 residents who experienced an integrated year were “better prepared” than those residents of previous classes [3]. Our paper could serve as an objective reference point of clinical ability for future studies that investigate the clinical preparedness of PGY-2 residents as all ophthalmology programs in the United States offer an integrated PGY-1 year.

There are several limitations within our study that are necessary to recognize. Our study presents a small sample size taken from a single institution from a single PGY-2 class. In addition, high-acuity cases were limited within the seven-week period with only a few cases for each diagnosis to evaluate resident performance. Our analysis is restricted by the number of participants (five PGY-2 residents) with variability in the number of consults seen by each resident within the seven-week period, prior exposure to ophthalmology, and variance in each resident’s internship training. The strength of our study is the consistent repeat evaluation of all consults by a senior resident over a seven-week period. With over 100 ophthalmology residency programs throughout the United States serving different populations, additional studies are needed to see if the population treated and the volume of consultations during an on-call period affect the time required to achieve 70% accuracy at the PGY-2 level. Limited knowledge paired with fatigue from a large volume of consultations each day may affect a resident’s diagnostic ability. A large-scale study could elucidate such differences in diagnostic accuracy over the numerous ophthalmology programs. The implementation of integrated internships in 2023 brought a new change with added ophthalmology exposure prior to beginning the PGY-2 transition, and this paper could serve as a benchmark for future studies to explore its effect on diagnostic accuracy.

## Conclusions

The transition to ophthalmology residency is challenging, especially given the expectations to provide accurate and appropriate care with minimal training and supervision. While a majority of programs utilize a buddy-call system and preparatory courses, there is no standardization of preparation that limits medical error and encourages autonomous decision-making in new ophthalmologists in training. Implementing buddy-call for three to four weeks increased diagnostic accuracy to over 70%. Increased exposure to ocular cases throughout the initial weeks of PGY-2 year, in addition to high-volume clinics, improved accuracy as well. High-acuity diagnoses were consistently correct 100% of the time from week five onward. Our analysis indicates that a five-week supervised PGY-2 call would provide an optimal time frame to ensure patient safety, promote autonomous decision-making, and decrease senior call burden.
